# Serum Concentration of Genistein, Luteolin and Colorectal Cancer Prognosis

**DOI:** 10.3390/nu11030600

**Published:** 2019-03-12

**Authors:** Ruijingfang Jiang, Gernot Poschet, Robert Owen, Muhabbet Celik, Lina Jansen, Rüdiger Hell, Michael Hoffmeister, Hermann Brenner, Jenny Chang-Claude

**Affiliations:** 1 Division of Cancer Epidemiology, German Cancer Research Center (DKFZ), 69120 Heidelberg, Germany; r.jiang@dkfz-heidelberg.de (R.J.); m.celik@dkfz-heidelberg.de (M.C.); 2 Medical Faculty Heidelberg, University of Heidelberg, 69120 Heidelberg, Germany; 3 Centre for Organismal Studies (COS), University of Heidelberg, 69120 Heidelberg, Germany; gernot.poschet@cos.uni-heidelberg.de (G.P.); ruediger.hell@cos.uni-heidelberg.de (R.H.); 4 Division of Preventive Oncology, National Center for Tumor Diseases, 69120 Heidelberg, Germany; R.Owen@dkfz-Heidelberg.de (R.O.); h.brenner@dkfz-heidelberg.de (H.B.); 5 Division of Clinical Epidemiology and Aging Research, German Cancer Research Center (DKFZ), 69120 Heidelberg, Germany; l.jansen@dkfz.de (L.J.); m.hoffmeister@dkfz.de (M.H.); 6 German Cancer Consortium (DKTK), German Cancer Research Center (DKFZ), 69120 Heidelberg, Germany; 7 University Cancer Center Hamburg (UCCH), University Medical Center Hamburg-Eppendorf, 20246 Hamburg, Germany

**Keywords:** colorectal cancer, phytoestrogens, genistein, luteolin, prognosis

## Abstract

Although flavonoid phytoestrogens have been suggested to be associated with reduced risk of colorectal cancer (CRC), their influence on CRC prognosis remains uncertain. A population-based cohort of 2051 patients diagnosed with stage I–III CRC in southwest Germany in 2003–2010 were followed for five years. Post-diagnostic serum concentration of genistein and luteolin were measured using Ultra-Performance Liquid Chromatography with mass spectrometry. Multivariable Cox regression analysis was conducted to calculate the Hazard Ratios (HRs) and 95% confidence interval (CI) for the association between flavonoids concentration and overall morality, CRC-specific mortality, CRC recurrence, and disease-free survival (DFS). Median (interquartile range) serum concentration of genistein and luteolin was 11.90 ng/µL (10.08–14.13) and 7.20 ng/µL (6.40–8.16), respectively. Neither serum genistein nor luteolin was associated with CRC prognosis. There was no clear evidence of departure from linearity. However, the association might be differential by adjuvant chemotherapy. Associations pointed towards lower risk in patients who received chemotherapy and higher risk in those without chemotherapy for overall mortality regarding serum genistein (P-interaction = 0.02) and correspondingly for CRC recurrence (P-interaction: 0.03) and DFS (P-interaction: 0.01) with respect to luteolin. Our study provides little evidence that serum genistein and luteolin are associated with colorectal cancer prognosis. Future studies are warranted to evaluate the potential interaction with adjuvant chemotherapy.

## 1. Introduction

Flavonoid phytoestrogens are bioactive, polyphenolic compounds found in plant-based food and share common chemical structural characteristics with endogenous estrogen. There are six subclasses of flavonoid phytoestrogen: Isoflavones, flavones, flavonols, flavanones, flavanols and anthocyanins. Major dietary sources of flavonoid phytoestrogens include fruits, vegetables, tea, wine, grains and herbs [1,2].

After consumption, flavonoids undergo extensive metabolism. Absorption occurs in both the small and large intestines, with a large portion of flavonoid metabolites reaching the colon [3]. Exposure to a substantial amount of flavonoids in the colon can thus play a role in the development of colorectal cancer. However, due to the limited surface for absorption in the colon, the absorption rate is very low and the urinary excretion rate of flavonoid subclasses ranges from 0.3–20% [4,5]. Both low oral bioavailability and low correlation between dietary intake and serum biomarker concentration have been observed in intervention studies [6,7].

Experimental studies have shown that flavonoid phytoestrogens have antioxidant, anti-inflammation and anti-cancer properties. Several biological mechanisms are involved in the anti-tumorigenic effect of flavonoids, including anti-angiogenesis, induction of apoptosis, inhibition of tumor cell adhesion and invasion, and estrogenic activities [8]. With a similar chemical structure to endogenous estrogen, phytoestrogens could bind to the estrogen receptors and activate estrogenic responsive pathways, and thereby reduce cell proliferation and differentiation in colon cancer cells [9]. The inverse association between menopausal hormone use and colorectal cancer (CRC) which indicates an involvement of estrogen exposure in colorectal carcinogenesis, also supports the hypothesis of a possible role of phytoestrogens in CRC development. Based on observational studies, a recent systematic review and meta-analysis found insufficient evidence for an association between phytoestrogens and colorectal cancer risk [10]. Yet dietary fiber intake from cereals and vegetables, which are flavonoid-rich foods, has been found to be associated with lower overall mortality and CRC-specific mortality [11]. In a randomized controlled trial in patients with colonic adenomas, short term supplementary of phytoestrogens resulted in increased ESR2 protein expression compared with the placebo group [12]. Positive tumoral ESR2 expression is associated with the grade and stage of CRC [13] and inversely associated with CRC progression [14]. Only one study has so far specifically investigated the association between dietary intake of flavonoid subclasses and CRC prognosis, and found no associations for isoflavones and flavones [15]. However, the study was based on a small number of samples. Assessment of association based on dietary intake is hampered by an incomprehensive database and the large inter-individual variation in bioavailability of flavonoids. Therefore, serum flavonoid metabolites may be a more accurate measurement of exposure than estimated dietary intake to assess the role of flavonoids. In the present study, we used serum biomarkers to quantitate the bioactive flavonoids, genistein for isoflavones and luteolin for flavones, and examined the association of genistein and luteolin with CRC prognosis in a prospective study with a large sample size.

## 2. Materials and Methods

### 2.1. Study Design and Study Population

The patient cohort is derived from the DACHS study (Darmkrebs: Chancen der Verhütung durch Screening) which is an ongoing population-based case-control study conducted in southwest Germany from 2003. Details of the design and methods were described previously [16,17]. Briefly, patients with a first diagnosis of invasive primary colorectal cancer (ICD-10: C18–C20) are eligible for recruitment if they are aged 30 or older and German-speaking. They were invited to participate in this study by their physician shortly after their diagnosis. 

Comprehensive data of the patients on sociodemographic, lifestyle and reproductive factors and family and medical histories were collected by a trained interviewer through standardized questionnaires at recruitment shortly after diagnosis. Clinical and pathological data were retrieved from the medical records. Three years after diagnosis, treatment details, disease progression and comorbidities information were obtained from their physicians with a standardized questionnaire. At the five-year follow-up, a self-administrated questionnaire was sent to each participant to update their information and collect further data on quality of life, and long-term outcomes including cancer recurrence and comorbidities. Vital status at both three-year follow-up and five-year follow-up was obtained from population registries, and the cause of death was verified by death certificates. This study was approved by the ethics committees of the Medical Faculty of Heidelberg University and the State Medical Boards of Baden-Württemberg and Rhineland-Palatinate. Written informed consent was obtained from all participants.

### 2.2. Serum Flavonoid Phytoestrogens Measurement

Serum concentrations of genistein and luteolin were measured by Ultra-Performance Liquid Chromatography with mass spectrometry (UPLC/MS) at the Centre for Organismal Studies (COS), University of Heidelberg over a period of six months. Blood samples were drawn at the time of the baseline interview shortly after diagnosis, from which multiple aliquots were made and stored at −80 °C until measurement. The extraction approach was adapted from a validated method [18,19]. Briefly, 108 µL sodium acetate buffer (0.14 M, pH 5) and 12 µL β-glucuronidase/aryl sulphatase from Helix pomatia-Sigma-Type HP-2 (5.0 mL/1205 units) were added to 100 µL serum to hydrolyze the phytoestrogen conjugates into aglycones. The phytoestrogen components were further extracted on a Strata-X SPE 96- well solid-phase extraction (SPE) plate (Phenomenex), dried under nitrogen and re-dissolved in methanol. The internal standards were added to each sample to account for the losses during the SPE. Quality control samples were treated the same way as study samples to assess the stability of measurement performance. There were two sources of quality control samples with different phytoestrogen concentration. One was from pooled serum samples from a blood bank which contained average phytoestrogen concentration, while the other was from pooled serum samples in a clinical trial on soy intake which contained high phytoestrogen concentration. In every batch, four quality control samples (two with high, and two with average phytoestrogen concentration) were measured. Phytoestrogen compounds were separated by reversed phase chromatography on an Acquity BEH C18 column (50 mm × 2.1 mm, 1.7 µm, Waters) and determined via mass spectrometry (Acquity QDa detector, Waters). Identification of separated phytoestrogens was based on their monoisotopic mass in negative-ion mode [M − H]^−^ with electrospray ionization at 0.8 kV capillary voltage and acquisition of individual single ion records using optimized cone voltages (CV): 269 *m*/*z* for genistein (CV 20 V) and 285 *m*/*z* for luteolin (CV 20 V). The limit of detection for genistein and luteolin was 0.25 pg/µL. 

Inter-batch variation was observed in our study, and the inter-batch coefficient of variation was 61.3% and 44.0% for genistein and luteolin, respectively. It may have been introduced by either the SPE extraction step or the UPLC measurement step. Therefore, a three-step re-calibration method was used to account for the batch effect [20]. First, a linear model was built with the concentration of flavonoid phytoestrogens as the dependent variable and the most relevant food source (grain intake for isoflavone and alcohol assumption for luteolin), timing of blood drawn with respect to surgery (before surgery, after surgery), indicator of preparation SPE plate and indicator of measurement batch as independent variables. Second, the average plate beta-coefficient and batch beta-coefficient was calculated by summing up the beta-coefficients of each SPE plate and each measurement batch, and then dividing by the number of plates and batches. The beta coefficient for each plate and batch was centered by subtracting the average of plate coefficients and batch coefficients. Finally, the phytoestrogen concentration was recalibrated by subtracting the sum of the mean-centered plate and batch beta-coefficient from the original concentration. Therefore, the recalibrated concentration accounted for the batch effect and the variation independent of main food source and timing of blood draw with respect to surgery between batches.

### 2.3. Tumor Tissue Analyses

The paraffin-embedded surgical specimens were requested from the corresponding department of pathology for a subsample of patients. The expression of estrogen receptor beta (ESR2) was determined by immunohistochemistry in the tissue microarray sections using the antibody 14C8 by Abcam (Toronto, ON, Canada) [14]. KRAS mutation was determined by single-stranded conformational polymorphism technique (SSCP) for most of the samples while Sanger sequencing was used for the remaining samples [21]. CpG island methylator phenotype (CIMP) was analyzed by methylation-specific PCR after bisulfite conversion. CIMP-negative, CIMP-low, CIMP-high was defined as hypermethylation at 0, 1–2, 3–5 loci out of 5 selected loci (MGMT, MLH1, MINT1, MINT2, and MINT31) [22].

### 2.4. Statistical Analysis

For this analysis, patients at stage I to III with available phytoestrogen measurement and recruited between January 2003 and December 2010 were included. Ten patients without surgery were further excluded from the analysis since surgery is the main treatment for stage I to III CRC and removal of intestine might influence the absorption of phytoestrogen. Multivariate Cox proportional hazard models accounting for late entry were used to evaluate the association of genistein and luteolin with prognosis of colorectal cancer, including overall mortality, CRC- specific mortality, CRC recurrence, and disease-free survival. Re-calibrated genistein and luteolin concentration were modeled both as a continuous variable after log2 transformation and in four categories defined by quartiles. In the latter models, the lowest quartile was used as the reference category. The follow-up period was defined as the interval between the date of study recruitment and the date of censor. Individuals were censored at the date of death, last contact or outcome of interest, whichever came first. Patients who were censored within 30 days after diagnosis were further excluded. Additional covariates were selected based on backward selection at the α-level of 0.2 from the variables including gender, stage, cancer site, body mass index (BMI) category, smoking, alcohol consumption, diabetes, cardiovascular disease (CVD), education, physical activity, detection mode, history of colonoscopy, regular use of nonsteroidal anti-inflammatory drugs, constipation, antibiotics use, red meat intake, adjuvant chemotherapy, radiotherapy, and timing of blood draw. After variable selection, age at diagnosis, gender (female, male), stage (I,II,III), cancer site (proximal colon, distal colon, rectal, unknown), BMI (<25, 25–<30, ≥30), education (low, intermediate, high), physical activity (<138.9, 138.9–<201.6, 201.6–<286.9, ≥286.9 MET-h/week), screening detected tumor (yes, no), adjuvant chemotherapy (yes, no), CVD (yes, no), diabetes (yes, no), constipation (yes, no), interval between chemotherapy and blood drawn (before/no chemotherapy, within chemotherapy, after chemotherapy), and interval between surgery and blood drawn (before surgery, after surgery) were included in the final model. The analyses were performed with complete cases after exclusion of participants with missing covariates (exclusion <3%). Proportional hazard assumptions were checked for all included variables, and no substantial departure was detected.

To investigate potential effect modification, subgroup analyses were conducted by demographic factors (age, gender), clinical factors (cancer site, stage), treatment factor (adjuvant chemotherapy), comorbidity-related factors (CVD, diabetes, BMI), tumor molecular characterization (KRAS, CIMP, ESR2 expression) and timing of blood draw (with respect to chemotherapy and diagnosis). Subgroup analyses by adjuvant chemotherapy were performed among those with a stage II or III CRC who had the possibility to undergo adjuvant chemotherapy according to the guidelines. Subgroup analyses by tumor molecular characterization were restricted to those for whom tumor tissue was available. The association analysis in female was further adjusted for hormone replacement therapy (ever, never). The interaction terms were evaluated by likelihood ratio test statistics, comparing models with and without interaction term. 

To account for the non-linear effect of genistein and luteolin, we further carried out a restricted cubic spline model with three fixed nodes (10%, 50% and 90%) [23], which corresponds to 8.75, 12.30, and 19.13 for genistein and 5.69, 7.39, and 9.76 for luteolin. The concentration of genistein and luteolin at the 10th percentile was taken as reference point. The cubic spline regression included all potential confounders mentioned above. Departure from linearity in this model was evaluated by using the likelihood ratio statistics to compare the model with and without non-linear terms. 

All analyses were performed using the SAS statistical software package, version 9.3 (SAS Institute, Cary, NC, USA). All tests were two-sided with a significant level of 0.05. 

## 3. Results

Of the 2051 patients diagnosed with stage of I to III colorectal cancer, serum genistein concentration was detected in 2029 participants, and luteolin concentration in 2011 participants. The mean age at diagnosis was 68.2 years (standard deviation: 10.6 years). Approximately 40% of the patients were female, and 59% had colon cancer. Blood was drawn shortly after diagnosis (median: 79 days, interquartile range (IQR): 14–285) and 93% were after surgery (median: 47 days, IQR: 10–267). Median (IQR) serum concentration of genistein and luteolin was 11.90 ng/µL (10.08–14.13) and 7.20 ng/µL (6.40–8.16), respectively. The genistein concentration was higher among participants with older age, a history of diabetes or cardiovascular disease and blood drawn before surgery, while luteolin concentration is higher among those with KRAS mutations (Table 1, Table S1). Overall, there were no significant differences across quartiles of either genistein or luteolin concentration according to gender, stage, CRC sites, grade, BMI, education, tumor detection mode, and adjuvant chemotherapy. 

After a median follow-up of 5.2 years (IQR: 4.4–5.3), a total of 475 patients died (23.2%), of whom 254 (12.5%) died of CRC. Besides, 400 (19.6%) patients had a CRC recurrence. In total, 585 (28.6%) had either deaths or recurrences as events.

**Figure 1:**
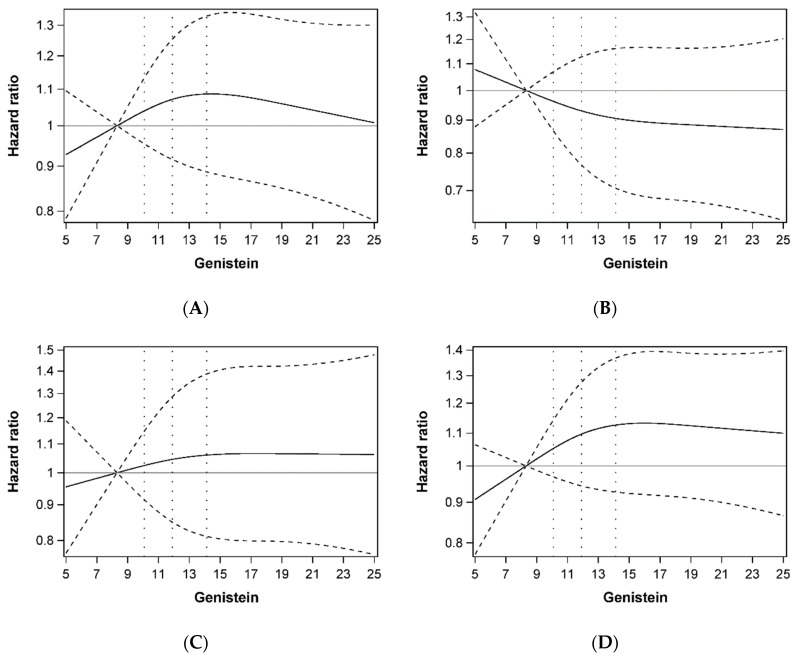
Dose-response relationship plots of the association between genistein concentration (ng/mL) and overall mortality (**A**), colorectal cancer specific mortality (**B**), colorectal cancer recurrence (**C**), and disease-free survival (**D**). Hazard ratios were estimated using restricted cubic-spline proportional hazard models with three knots placed at the 10th, 50th, and 90th percentiles of genistein concentration. The 10th percentile of genistein concentration was treated as the reference level, and 25th, 50th, and 75th percentile were shown as dashed vertical lines.

**Figure 2:**
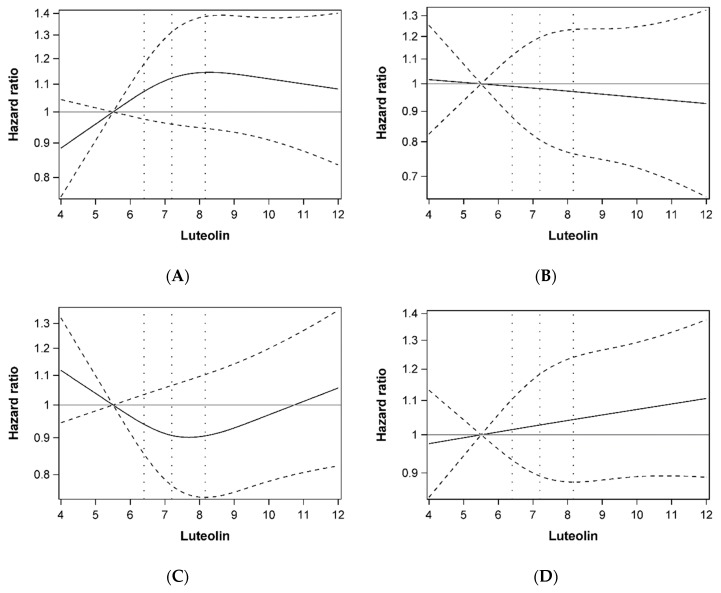
Dose-response relationship plots of the association between luteolin concentration (ng/mL) and overall mortality (**A**), colorectal cancer specific mortality (**B**), colorectal cancer recurrence (**C**), and disease-free survival (**D**). Hazard ratios were estimated using restricted cubic-spline proportional hazard models with three knots placed at the 10th, 50th, and 90th percentiles of luteolin concentration. The 10th percentile of luteolin concentration was treated as the reference level, and 25th, 50th, and 75th percentile were shown as dashed vertical lines.

Multivariable-adjusted HRs for genistein and luteolin concentration in relation to colorectal cancer prognosis are shown in Table 2. Compared with the lowest quartile, the highest quartile of serum genistein concentration was not associated with the risk of overall mortality (HR: 1.00, 95% CI: 0.77–1.30), CRC-specific mortality (HR: 0.83, 95% CI: 0.58–1.19), CRC recurrence (HR: 0.98, 95% CI: 0.72–1.34), and disease-free survival (HR: 1.03, 95% CI: 0.80–1.32) in patients with stage I-III CRC. Similar to genistein, serum luteolin concentration was not associated with overall mortality (HR: 1.19, 95% CI: 0.92–1.53), CRC-specific mortality (HR: 1.05, 95% CI: 0.74–1.47), CRC recurrence (HR: 1.02, 95% CI: 0.76–1.36), or disease-free survival (HR: 1.14, 95% CI: 0.90–1.44) (Table 2). No clear evidence of departure from linearity was detected for the association of genistein and luteolin and colorectal cancer prognosis (Figure 1 and Figure 2).

The associations of genistein and luteolin with colorectal cancer prognosis were not modified by age, gender, cancer site, stage, BMI, diabetes, and CVD. However, a statistically significant interaction with adjuvant chemotherapy was observed for both genistein and luteolin among stage II and stage III patients. Serum genistein was associated with better overall survival among those with adjuvant chemotherapy (Q4 vs. Q1 HR: 0.68, 95% CI: 0.44–1.04), but with poorer overall survival among those without adjuvant chemotherapy (Q4 vs. Q1 HR: 1.47, 95% CI: 0.99–2.17, P-interaction = 0.02) (Table S2). For luteolin, higher serum luteolin concentration was associated with a reduced recurrence in the patients with adjuvant chemotherapy (Q4 vs. Q1 HR: 0.67, 95% CI: 0.44–1.02) and an increased recurrence among those without adjuvant chemotherapy (Q4 vs. Q1 HR: 1.60, 95% CI: 1.01–2.55, P-interaction: 0.03). A similar association of luteolin was observed for disease-free survival (P-interaction: 0.01), and the corresponding HRs (95% CI) for patients with and without adjuvant chemotherapy were 0.75 (0.52–1.10) and 1.53 (1.06–2.21) (Table S3).

No substantial differences were found according to the timing of blood drawn with respect to diagnosis and chemotherapy for both genistein and luteolin (Tables S4 and S5). The highest quartile of genistein was associated with significantly improved CRC-specific mortality among patients with blood draw within one to six months after diagnosis (HR: 0.38, 95% CI: 0.17–0.87) but without evidence of differential effect (P-interaction = 0.32).

Analyses by colorectal cancer molecular features including CIMP status, MSI status, KRAS mutation and ESR2 expression showed no effect heterogeneity for either genistein or luteolin (Tables S6 and S7). The highest quartile of serum luteolin concentration compared to the lowest quartile was associated with a poorer overall mortality (HR: 2.76, 95% CI: 1.25–6.10, P-interaction: 0.58) and disease-free survival among CIMP high CRC patients (HR: 2.52, 95% CI: 1.18–5.41, P-interaction = 0.49), but the associations were not substantially differential by CIMP status.

## 4. Discussion

In this large population-based prospective study of colorectal cancer patients, no significant association was found for either serum genistein or luteolin concentration with overall mortality, CRC-specific mortality, CRC recurrence, or disease-free survival. However, there may be differential association according to adjuvant chemotherapy.

The results from this study were in line with a previous study of 411 CRC patients conducted in Spain, which is by far the only study that investigated a possible association between colorectal cancer prognosis and phytoestrogen exposure although based on estimated dietary intake. They did not find dietary intake of flavone and isoflavone to be associated with either colorectal cancer recurrence or overall mortality [15]. Compared with that study, the present study has a larger sample size (2051 versus 411), patients of similar age at CRC diagnosis (68.2 versus 67.0), a shorter follow-up (5.2 versus 8.6 years), and used a different exposure measurement (post-diagnostic serum biomarker concentration versus estimated pre-diagnostic dietary intake).

Our evaluation of potential effect modification for several factors suggest that the association between serum flavonoid phytoestrogens concentration and colorectal cancer prognosis might be differential by adjuvant chemotherapy. Higher serum genistein and luteolin concentrations appeared to be associated with an improved CRC prognosis among patients who had undergone adjuvant chemotherapy and with a poorer prognosis among those who had not, although not all the associations were statistically significant. While this might be a chance finding due to multiple testing, there is some biological plausibility for a beneficial effect of high phytoestrogen levels in patients who received adjuvant chemotherapy. Genistein and luteolin have been found to sensitize chemo-resistant colorectal cancer cells to chemotherapy using 5-fluorouracil [24,25], which is the predominant first-line chemotherapy for colorectal cancer patients. Therefore, genistein and luteolin could enhance the efficacy of chemotherapy. The adverse effect of genistein and luteolin on the long-term outcome of CRC patients without adjuvant chemotherapy may be due to the factors resulting in undertreatment, such as old age, comorbidities [26,27,28]. Indeed, elderly patients and those with a CVD history in this study had a higher level of genistein concentration. Although age and comorbidities such as CVD, diabetes were adjusted for in the analyses, residual confounding may still exist. Therefore, this exploratory finding requires confirmation in further studies.

Our study did not support a significant association of genistein and luteolin with colorectal cancer prognosis, yet anticarcinogenic effects of genistein and luteolin have been found in experimental studies, particularly mediated through an estrogenic effect. Owing to a similar chemical structure as endogenous estrogen, flavonoid phytoestrogens can bind to ESR2, the predominant estrogen receptor expressed in the colon mucosa, and activate estrogen-responsive pathways [29,30], which play an important role in tumorigenesis and progression of colorectal cancer [31,32]. Genistein has been found to reduce proliferation of colon cancer cells with high expression of ESR2 and the anti-proliferation effect of genistein depends on binding and activation of ESR2 [33]. Flavones had been reported both as an agonist and antagonist of the estrogen receptor [34,35], and may regulate cell proliferation and differentiation through estrogen-dependent pathways [36]. We examined, but did not find statistically significant effect modification by ESR2 expression in the tumor. In our study, we used the 14C8 (Abcam) as the ESR2 antibody which is a commonly used antibody in research. However, recent publications indicate that this antibody is not sufficiently specific and binds to additional proteins [37,38], which may introduce irreproducibility and warrants attention. Due to the variability of the performance of the antibody and the lack of significant effect modification, evidence is too limited to draw conclusions with respect to possible effect heterogeneity by ESR2 expression.

In the present study, flavonoid phytoestrogen levels were quantified by the UPLC/MS method which was adapted from a previously validated method [18,19]. In contrast to dietary intake estimated by food frequency questionnaire, serum biomarkers are more accurate, objective and sensitive for quantification of bioactive metabolites of flavonoid phytoestrogen which have undergone extensive metabolism. After consumption, phytoestrogen metabolites are first absorbed in the small intestine [39] and the unabsorbed metabolites will be further absorbed in the colon after deglycosylation by colon microflora [40]. Therefore, gut microflora, gut transit-time, treatment and lifestyle factors in addition to dietary intake may lead to intra- and inter-individual variation of serum phytoestrogen concentration and should be addressed in recalibration and modeling steps, as was performed in this study. In Western populations, the main food source of genistein is processed food containing soy additives, such as bread, bakery products, cereal and processed meat [41,42,43], and for luteolin mainly beverages, such as alcohol [44]. Whole grain food intake and alcohol was included in the recalibration model for genistein and luteolin, respectively. Antibiotic use has been shown to influence the gut microflora and reduce the phytoestrogen concentration for up to 16 months [45]. In our study, only patients who had undergone surgery were included. Perioperative antibiotic prophylaxis is routinely administered in CRC patients within one hour before surgery and colon removal will influence colonic absorption. Therefore, whether the blood was drawn before or after surgery was an important factor influencing phytoestrogen concentrations and was thus considered for both re-calibration and adjustment. Apart from surgery, chemotherapy could induce disturbed homeostasis of gut microflora [46,47]; therefore the timing of blood draw with respect to chemotherapy was also accounted for in the analyses. Constipation could slow down intestinal transit and be indicative of the severity of colorectal cancer; therefore it could influence serum phytoestrogen concentration in both directions [48]. Since detailed information on constipation status was not available, laxative use was used as a proxy for constipation status.

The strengths of our study include the prospective study design; a large number of colorectal cancer patients; the completeness of follow-up; and a validated method [18,19] to measure the bioactive flavonoid phytoestrogen metabolites. We also have comprehensive information on important risk factors and prognostic factors of CRC to adjust for confounders and assess potential effect modification. Our study has several limitations. First, a single measurement of serum concentration of phytoestrogen may only capture short term exposure to phytoestrogen shortly after diagnosis. Since CRC patients were recruited shortly after diagnosis and mainly after surgery, the phytoestrogen levels are likely to be influenced by the diagnosis and treatment. Additionally, participants might still be under hospital diet when the blood was drawn and would probably return to their own dietary habit afterward. Therefore, we adjusted for, and conducted stratified analyses by the timing of blood draw with respect to diagnosis and treatment. Although phytoestrogen concentration after adopting the patient’s normal diet may be more relevant to assess the association with long-term prognosis, we did not find any significant interaction between the timing of blood drawn and phytoestrogen concentration with respect to CRC prognosis. Thus, a single measurement shortly after diagnosis might not have severely biased the association. Second, although the recalibration method corrected for the batch effect and generated a comparable distribution of serum phytoestrogen across batches, it reduced the inter-individual variation of serum phytoestrogens. Therefore, it may have led to an underestimation of the true association of serum genistein and luteolin with colorectal cancer prognosis. Furthermore, serum phytoestrogen may be associated with other unmeasured factors, which were not accounted for in the current recalibration approach. However, demographic and lifestyle factors have been found to explain only 2% of the variation of phytoestrogen concentration [42], so that even if unaccounted for they are not likely to greatly influence the association estimates. Finally, power is limited for some of the subgroup analyses and association assessment for serum luteolin. Due to the low absorption capacity of luteolin [5], the range of serum luteolin concentration is relatively low in our study population. A larger number of samples would be needed to capture an association with such a small variance of exposure levels.

In conclusion, our study provides limited evidence of an association between post-diagnostic serum genistein and luteolin and the prognosis of colorectal cancer. However, higher genistein and luteolin concentrations may possibly be associated with a better prognosis among those who underwent adjuvant chemotherapy. Future studies with the blood sample drawn and phytoestrogen measured at different time-points and larger sample size are required to confirm the current findings.

## Figures and Tables

**Table 1:** Characteristics of CRC patients by quartiles of serum genistein and luteolin concentrations ^1^.

	Genistein ^2^ (N = 2029)				Luteolin ^3^ (N = 2011)			
	Q1	Q2	Q3	Q4	Q1	Q2	Q3	Q4
No. of patients	508	507	507	507	503	503	503	502
Age mean (sd)	67.7 (9.9)	67.6 (10.5)	67.7 (11.0)	69.7 (11.0)	68.6 (10.2)	68.2 (10.8)	67.5 (10.9)	68.6 (10.7)
Gender								
Male	311 (61.2)	311 (61.3)	292 (57.6)	294 (58.0)	303 (60.2)	295 (58.7)	303 (60.2)	292 (58.2)
Female	197 (38.8)	196 (38.7)	215 (42.4)	213 (42.0)	200 (39.8)	208 (41.3)	200 (39.8)	210 (41.8)
Stage								
1	157 (30.9)	121 (23.9)	122 (24.1)	107 (21.1)	135 (26.8)	117 (22.6)	126 (25.0)	128 (25.5)
2	156 (30.7)	202 (39.8)	179 (35.3)	205 (40.4)	171 (34.0)	199 (39.7)	183 (37.4)	179 (35.7)
3	195 (38.4)	184 (36.3)	206 (40.6)	195 (38.5)	197 (39.2)	187 (37.7)	194 (38.6)	195 (38.8)
Cancer site								
Proximal colon	144 (28.3)	166 (32.7)	158 (31.2)	182 (35.9)	155 (30.8)	174 (34.6)	161 (32.0)	157 (31.3)
Distal Colon	129 (25.4)	142 (28.0)	135 (26.6)	126 (24.9)	137 (27.2)	124 (24.6)	134 (26.6)	138 (27.5)
Rectum	234 (46.1)	198 (38.1)	211 (41.6)	198 (39.0)	209 (41.6)	203 (40.4)	208 (41.4)	205 (40.8)
Other	1 (0.2)	1 (0.2)	3 (0.6)	1 (0.2)	2 (0.4)	2 (0.4)	0 (0)	2 (0.4)
BMI								
<25	171 (33.6)	214 (42.2)	175 (34.5)	198 (39.1)	194 (38.6)	186 (37.0)	186 (37.0)	188 (37.5)
25–<30	232 (45.7)	212 (41.8)	228 (45.0)	209 (41.2)	217 (43.1)	220 (43.7)	211 (41.9)	223 (44.4)
≥30	105 (20.7)	81 (16.0)	104 (20.5)	100 (19.7)	92 (18.3)	97 (19.3)	106 (21.1)	91 (18.1)
Education								
Low	307 (60.4)	300 (59.2)	314 (61.9)	326 (64.3)	315 (62.6)	299 (59.4)	309 (61.4)	316 (63.0)
Intermediate	118 (23.2)	108 (21.3)	108 (21.3)	113 (22.3)	109 (21.7)	113 (22.5)	115 (22.9)	105 (20.9)
High	83 (16.4)	99 (19.5)	85 (16.8)	68 (13.4)	79 (15.7)	91 (18.1)	79 (15.7)	81 (16.1)
Physical activity (MET-h/week)								
<138.9	118 (23.2)	117 (23.1)	129 (25.4)	139 (27.4)	118 (23.4)	131 (26.0)	110 (21.9)	133 (26.5)
138.9–<201.6	114 (22.4)	131 (25.8)	124 (24.5)	137 (27.0)	114 (22.7)	114 (22.7)	143 (28.4)	134 (26.7)
201.6–<286.9	136 (26.8)	133 (26.2)	130 (25.6)	109 (21.5)	126 (25.1)	128 (25.5)	136 (27.0)	117 (23.3)
≥286.9	140 (27.6)	126 (24.9)	124 (24.5)	122 (24.1)	145 (28.8)	130 (25.8)	114 (22.7)	118 (23.5)
Screening detected cancer								
No	367 (72.2)	401 (79.1)	366 (72.2)	377 (74.4)	371 (73.8)	365 (72.6)	379 (75.4)	378 (75.3)
Yes	141 (27.8)	106 (20.9)	141 (27.8)	130 (25.6)	132 (26.2)	138 (27.4)	124 (24.6)	124 (24.7)
Chemotherapy								
No	335 (65.9)	321 (63.3)	315 (62.1)	323 (63.7)	326 (64.8)	323 (64.2)	308 (61.2)	327 (65.1)
Yes	173 (34.1)	186 (36.7)	192 (37.9)	184 (36.3)	177 (35.2)	180 (35.8)	195 (38.8)	175 (34.9)
Diabetes								
No	413 (81.3)	417 (82.3)	409 (80.7)	388 (76.5)	395 (78.5)	411 (81.7)	415 (82.5)	391 (77.9)
Yes	95 (18.7)	90 (17.7)	98 (19.3)	119 (23.5)	108 (21.3)	92 (18.3)	88 (17.5)	111 (22.1)
CVD								
No	387 (76.2)	404 (79.7)	370 (73.0)	361 (71.2)	375 (74.5)	385 (76.5)	373 (74.2)	376 (74.9)
Yes	121 (23.8)	103 (20.3)	137 (27.0)	146 (28.8)	128 (25.5)	118 (23.5)	130 (25.8)	126 (25.1)
Constipation								
No	474 (93.3)	470 (92.7)	478 (94.3)	476 (93.9)	469 (93.2)	466 (92.6)	475 (94.4)	470 (93.6)
Yes	34 (6.7)	37 (7.3)	29 (5.7)	31 (6.1)	34 (6.8)	37 (7.4)	28 (5.6)	32 (6.4)
ESR2 status ^4^								
Negative	151 (44.7)	157 (43.3)	162 (47.1)	165 (47.8)	174 (49.0)	151 (42.5)	152 (45.9)	155 (45.6)
Positive	187 (55.3)	206 (56.7)	182 (52.9)	180 (52.2)	181 (51.0)	204 (57.5)	179 (54.1)	185 (54.4)
CIMP ^5^								
Negative/Low	324 (85.7)	338 (82.6)	335 (82.5)	317 (80.7)	347 (86.3)	323 (80.1)	304 (79.8)	331 (85.1)
High	54 (14.3)	71 (17.4)	71 (17.5)	76 (19.3)	55 (13.7)	80 (19.9)	77 (20.2)	58 (14.9)
KRAS mutation ^6^								
Wild type	236 (67.2)	266 (69.6)	273 (70.5)	232 (62.9)	253 (67.8)	265 (69.5)	252 (68.5)	232 (65.2)
Mutant	115 (32.8)	116 (30.4)	114 (29.5)	137 (37.1)	120 (32.2)	116 (30.5)	116 (31.5)	124 (34.8)
Interval between diagnosis and blood drawn								
<1 month	201 (39.6)	218 (43.0)	218 (43.0)	207 (40.8)	229 (45.5)	204 (40.6)	186 (37.0)	215 (42.8)
1–6 months	114 (22.4)	116 (22.9)	123 (24.3)	115 (22.7)	92 (18.3)	129 (25.6)	142 (28.2)	99 (19.7)
>6 months	193 (38.0)	173 (34.1)	166 (32.7)	185 (36.5)	182 (36.2)	170 (33.8)	175 (34.8)	188 (37.5)
Interval between surgery and blood drawn								
Before surgery	32 (6.3)	17 (3.4)	40 (7.9)	43 (8.5)	36 (7.2)	33 (6.6)	29 (5.8)	33 (6.6)
After surgery	476 (93.7)	490 (96.6)	467 (92.1)	464 (91.5)	467 (92.8)	470 (93.4)	474 (94.2)	469 (93.4)
Interval between chemotherapy and blood drawn								
Before/no chemo	420 (82.7)	404 (79.7)	397 (78.3)	396 (78.1)	405 (80.5)	408 (81.1)	390 (77.5)	398 (79.3)
During chemo	25 (4.9)	48 (9.5)	44 (8.7)	33 (6.5)	28 (5.6)	38 (7.6)	51 (10.2)	32 (6.4)
After chemo	63 (12.4)	55 (10.8)	66 (13.0)	78 (15.4)	70 (13.9)	57 (11.3)	62 (12.3)	72 (14.3)

^1^ All categorical variables were present as N (%); ^2^ Quartiles of serum genistein concentration: Q1, <10.08 ng/µL; Q2, 10.08–<11.90 ng/µL; Q3, 11.90–<14.13 ng/µL; Q4, ≥14.13 ng/µL; ^3^ Quartiles of serum luteolin concentration: Q1, <6.40 ng/µL; Q2, 6.40–<7.20 ng/µL; Q3, 7.20–<8.16 ng/µL; Q4, ≥8.16 ng/µL; ^4^ Data missing for 639 patients (genistein); data missing for 630 patients (luteolin).; ^5^ Data missing for 443 patients (genistein); data missing for 437 patients (luteolin); ^6^ Data missing for 540 patients (genistein); data missing for 533 patients (luteolin).; Abbreviation: BMI: Body mass index; CVD: Cardiovascular disease; ESR2: Estrogen receptor beta; CIMP: CpG island methylator phenotype.

**Table 2:** Association of serum genistein and luteolin concentrations with long-term outcomes in CRC patients ^1^.

	Overall Mortality		CRC-Specific Mortality ^2^		CRC-Recurrence ^3^		Disease-Free Survival ^4^	
	N	HR (95% CI)	N	HR (95% CI)	N	HR (95% CI)	N	HR (95% CI)
Genistein								
Q1	114	1.00 (Ref)	69	1.00 (Ref)	97	1.00 (Ref)	138	1.00 (Ref)
Q2	119	1.01 (0.78–1.31)	69	1.01 (0.72–1.42)	104	1.10 (0.81–1.49)	147	1.05 (0.82–1.34)
Q3	115	0.99 (0.77–1.29)	56	0.79 (0.55–1.13)	97	1.02 (0.76–1.38)	144	1.08 (0.84–1.38)
Q4	122	1.00 (0.77–1.30)	58	0.83 (0.58–1.19)	99	0.98 (0.72–1.34)	150	1.03 (0.80–1.32)
Linear	470	1.03 (0.90–1.19)	252	0.96 (0.80–1.15)	397	1.05 (0.89–1.25)	579	1.08 (0.94–1.24)
Luteolin								
Q1	117	1.00 (Ref)	71	1.00 (Ref)	108	1.00 (Ref)	148	1.00 (Ref)
Q2	116	1.12 (0.87–1.46)	64	0.98 (0.70–1.38)	90	0.82 (0.61–1.11)	134	0.95 (0.74–1.21)
Q3	110	1.07 (0.82–1.39)	50	0.77 (0.53–1.12)	92	0.80 (0.58–1.09)	140	0.97 (0.75–1.24)
Q4	125	1.19 (0.92–1.53)	65	1.05 (0.74–1.47)	104	1.02 (0.76–1.36)	154	1.14 (0.90–1.44)
Linear	468	1.12 (0.89–1.40)	250	0.96 (0.70–1.32)	394	0.99 (0.75–1.30)	576	1.09 (0.87–1.35)

^1^ Late-entry models were used after adjusting for age, gender (female, male), stage (I, II, III), cancer site (proximal colon, distal colon, rectal, unknown), BMI (<25, 25–<30, ≥30), education (low, intermediate, high), physical activity quartiles, screening detected tumor (yes, no), chemotherapy (yes, no), diabetes (yes, no), CVD (yes, no), constipation (yes, no), interval between chemotherapy and blood drawn (before/no chemotherapy, within chemotherapy, after chemotherapy), interval between surgery and blood drawn (before surgery, after surgery); ^2^ data missing for 14 patients; ^3^ data missing for 8 patients, 53 patients had an event before study entry; ^4^ data missing for 8 patients, 53 patients had an event before study entry; abbreviation: HR: Hazard ratio; CI: Confidence intervals; Ref.: Reference; BMI: Body mass index; CVD: Cardiovascular disease.

## Supplementary Materials

The following are available online at https://www.mdpi.com/2072-6643/11/3/600/s1, Table S1: Genistein and luteolin concentration by patient characteristics, Table S2: Association between genistein and colorectal cancer prognosis according to different subgroups, Table S3: Association between luteolin and colorectal cancer prognosis according to different subgroups, Table S4: Association of serum genistein and colorectal cancer prognosis by timing of blood drawn, Table S5: Association of serum luteolin and colorectal cancer prognosis by timing of blood drawn, Table S6: Association between genistein and colorectal cancer prognosis by tumoral molecular characterization.
