# Supplementary material for: Serum Concentration of Genistein, Luteolin and Colorectal Cancer Prognosis
Source: Nutrients. 2019 Mar 12;11(3):600. doi: 10.3390/nu11030600 (PMC6472030; doi:10.3390/nu11030600)
Supplement: Supplementary file 1 [file nutrients-11-00600-s001.pdf]

Table S1. Genistein and luteolin concentration by patient characteristics.

	Genistein			Luteolin		
	N	Median	IQR	N	Median	IQR
Age category						
<70	1063	11.74	10.00-13.79	1051	7.24	6.40-8.14
≥70	966	12.06	10.18-14.43	960	7.15	6.40-8.20
Gender						
Male	1208	12.05	10.18-14.23	1193	7.20	6.42-8.19
Female	821	11.73	10.05-14.00	818	7.19	6.39-8.14
Stage						
1	507	11.57	9.63-13.55	506	7.22	6.35-8.18
2	742	11.99	10.36-14.42	732	7.19	6.49-8.11
3	780	12.00	10.08-14.13	773	7.21	6.38-8.18
Cancer site						
Proximal colon	650	12.06	10.23-14.64	647	7.18	6.41-8.15
Distal Colon	532	11.81	10.21-13.91	533	7.24	6.38-8.21
Rectum	841	11.76	9.88-13.94	825	7.20	6.40-8.11
Other	6	13.19	11.21-13.83	6	6.77	6.08-8.80
BMI						
<25	758	11.85	10.25-14.31	754	7.19	6.38-8.14
25-<30	881	11.87	10.01-13.90	871	7.19	6.40-8.19
≥30	390	12.05	10.01-14.20	386	7.25	6.47-8.14
Education						
Low	1247	11.98	10.10-14.25	1239	7.23	6.38-8.19
Intermediate	447	11.81	9.97-14.14	442	7.20	6.41-8.14
High	335	11.65	10.08-13.52	330	7.15	6.45-8.13
Physical activity (MET-h/week)						

<138.9	503	12.08	10.20-14.45	492	7.18	6.43-8.25
138.9-<201.6	506	11.99	10.30-14.55	505	7.33	6.50-8.27
201.6- <286.9	508	11.65	9.93-13.59	507	7.18	6.40-8.06
≥286.9	512	11.74	9.83-13.92	507	7.06	6.28-8.05
Screening detected cancer						
No	1511	11.81	10.12-14.13	1493	7.24	6.41-8.18
Yes	518	12.01	9.90-14.14	518	7.11	6.39-8.13
Chemotherapy						
No	1294	11.80	10.01-14.13	1284	7.19	6.39-8.18
Yes	735	11.98	10.15-14.14	727	7.24	6.41-8.13
Diabetes						
No	1627	11.81	10.07-13.97	1612	7.20	6.41-8.14
Yes	402	12.09	10.17-14.75	399	7.19	6.33-8.29
CVD						
No	1522	11.74	10.06-13.90	1509	7.19	6.41-8.16
Yes	507	12.30	10.17-14.64	502	7.26	6.38-8.17
Constipation						
No	1898	11.92	10.08-14.14	1880	7.21	6.40-8.16
Yes	131	11.63	10.01-13.97	131	7.04	6.33-8.16
ESR2 status[1]						
Negative	635	11.97	10.13-14.23	632	7.16	6.29-8.15
Positive	755	11.71	10.08-13.99	749	7.15	6.44-8.15
CIMP[2]						
Negative/ Low	1314	11.88	10.08-13.99	1304	7.15	6.35-8.19
High	272	12.19	10.46-14.47	270	7.20	6.59-7.95
KRAS[3]						
Wild type	1007	11.91	10.18-13.88	1002	7.15	6.39-8.07
Mutant	482	12.06	10.18-14.57	476	7.22	6.40-8.23

Interval between diagnosis and blood drawn						
<1 month	844	11.92	10.18-14.06	834	7.14	6.33-8.22
1-6 months	468	12.04	10.10-14.09	462	7.26	6.60-8.01
>6 months	717	11.85	9.94-14.21	715	7.24	6.39-8.23
Interval between surgery and blood drawn						
Before surgery	132	12.62	10.32-15.03	131	7.03	6.35-8.16
After surgery	1897	11.81	10.07-14.05	1880	7.20	6.40-8.16
Timing between chemotherapy and blood drawn						
Before/no chemotherapy	1617	11.76	10.04-14.05	1601	7.17	6.39-8.16
During chemotherapy	150	12.00	10.48-13.52	149	7.32	6.65-8.05
After chemotherapy	262	12.37	10.17-14.64	261	7.26	6.30-8.37

1. Data missing for 639 patients (genistein); data missing for 630 patients (luteolin).
2. Data missing for 443 patients (genistein); data missing for 437 patients (luteolin).
3. Data missing for 540 patients (genistein); data missing for 533 patients (luteolin).

Abbreviation: IQR: interquartile range; MET, metabolic equivalent of task; BMI: body mass index; CVD: cardiovascular disease; ESR2: estrogen receptor beta; CIMP: CpG island methylator phenotype.

Table S2. Association between genistein and colorectal cancer prognosis according to different subgroups[1].

	Overall mortality		CRC-specific mortality		CRC-recurrence		Disease-free survival	
	HR (95% CI)	p_{int}	HR (95% CI)	p_{int}	HR (95% CI)	p_{int}	HR (95% CI)	p_{int}
Age		0.09		0.32		0.28		0.09
<70								
Q1	1.00 (Ref)		1.00 (Ref)		1.00 (Ref)		1.00 (Ref)	
Q2	1.28 (0.85 - 1.94)		1.14 (0.69 - 1.88)		1.35 (0.88 - 2.08)		1.40 (0.96 - 2.05)	
Q3	0.83 (0.53 - 1.30)		0.64 (0.37 - 1.12)		1.00 (0.64 - 1.55)		1.07 (0.72 - 1.58)	
Q4	0.92 (0.59 - 1.45)		0.63 (0.36 - 1.13)		0.99 (0.62 - 1.58)		1.10 (0.74 - 1.66)	
Linear	0.90 (0.69 - 1.19)		0.80 (0.57 - 1.14)		1.04 (0.79 - 1.37)		1.06 (0.83 - 1.34)	
≥70								
Q1	1.00 (Ref)		1.00 (Ref)		1.00 (Ref)		1.00 (Ref)	
Q2	0.83 (0.59 - 1.18)		0.85 (0.52 - 1.36)		0.81 (0.52 - 1.25)		0.80 (0.57 - 1.12)	
Q3	1.10 (0.80 - 1.53)		0.89 (0.55 - 1.44)		0.99 (0.65 - 1.51)		1.07 (0.78 - 1.47)	
Q4	1.07 (0.77 - 1.47)		0.93 (0.59 - 1.48)		0.92 (0.60 - 1.40)		0.99 (0.72 - 1.36)	
Linear	1.12 (0.94 - 1.32)		1.05 (0.84 - 1.31)		1.08 (0.87 - 1.34)		1.12 (0.94 - 1.33)	
Gender		0.51		0.83		0.88		0.47
Male								
Q1	1.00 (Ref)		1.00 (Ref)		1.00 (Ref)		1.00 (Ref)	

Q2	0.94 (0.68 - 1.30)	0.97 (0.61 - 1.53)	1.07 (0.73 - 1.58)	0.98 (0.72 - 1.33)
Q3	0.93 (0.66 - 1.31)	0.69 (0.42 - 1.16)	0.91 (0.60 - 1.37)	0.95 (0.69 - 1.30)
Q4	0.84 (0.60 - 1.19)	0.71 (0.43 - 1.17)	0.94 (0.62 - 1.42)	0.90 (0.66 - 1.24)
Linear	0.99 (0.83 - 1.20)	0.93 (0.71 - 1.22)	1.11 (0.87 - 1.41)	1.08 (0.89 - 1.30)
Female				
Q1	1.00 (Ref)	1.00 (Ref)	1.00 (Ref)	1.00 (Ref)
Q2	1.10 (0.71 - 1.71)	0.98 (0.58 - 1.67)	1.08 (0.66 - 1.79)	1.16 (0.75 - 1.80)
Q3	1.08 (0.70 - 1.66)	0.90 (0.53 - 1.52)	1.21 (0.75 - 1.94)	1.32 (0.87 - 2.00)
Q4	1.23 (0.81 - 1.87)	0.96 (0.57 - 1.61)	1.00 (0.61 - 1.62)	1.21 (0.80 - 1.82)
Linear	1.07 (0.84 - 1.35)	0.93 (0.69 - 1.25)	0.93 (0.71 - 1.22)	1.04 (0.83 - 1.31)
Site	0.72	0.48	0.46	0.84
Proximal colon				
Q1	1.00 (Ref)	1.00 (Ref)	1.00 (Ref)	1.00 (Ref)
Q2	1.23 (0.76 - 2.00)	0.89 (0.46 - 1.75)	0.70 (0.39 - 1.25)	1.04 (0.66 - 1.63)
Q3	1.15 (0.70 - 1.90)	0.90 (0.45 - 1.80)	0.85 (0.48 - 1.51)	1.04 (0.66 - 1.65)
Q4	1.35 (0.85 - 2.15)	1.07 (0.56 - 2.07)	0.93 (0.53 - 1.62)	1.19 (0.77 - 1.85)
Linear	1.23 (0.93 - 1.63)	1.29 (0.86 - 1.95)	1.26 (0.89 - 1.79)	1.23 (0.94 - 1.60)
Distal colon				

Q1	1.00 (Ref)		1.00 (Ref)		1.00 (Ref)		1.00 (Ref)	
Q2	0.91 (0.51 - 1.60)		0.92 (0.45 - 1.89)		1.09 (0.59 - 2.03)		1.01 (0.60 - 1.70)	
Q3	0.78 (0.44 - 1.40)		0.33 (0.13 - 0.79)		0.81 (0.42 - 1.55)		1.07 (0.64 - 1.80)	
Q4	0.74 (0.41 - 1.34)		0.46 (0.20 - 1.07)		0.63 (0.31 - 1.28)		0.83 (0.48 - 1.44)	
Linear	0.97 (0.62 - 1.52)		0.63 (0.32 - 1.21)		0.72 (0.43 - 1.20)		0.95 (0.64 - 1.41)	
Rectal								
Q1	1.00 (Ref)		1.00 (Ref)		1.00 (Ref)		1.00 (Ref)	
Q2	0.95 (0.64 - 1.41)		1.09 (0.66 - 1.80)		1.34 (0.86 - 2.10)		1.09 (0.75 - 1.59)	
Q3	0.99 (0.68 - 1.44)		0.94 (0.57 - 1.54)		1.17 (0.76 - 1.82)		1.06 (0.74 - 1.52)	
Q4	0.91 (0.61 - 1.36)		0.85 (0.50 - 1.45)		1.13 (0.71 - 1.80)		0.96 (0.65 - 1.41)	
Linear	0.96 (0.80 - 1.15)		0.90 (0.72 - 1.13)		1.03 (0.82 - 1.30)		1.02 (0.84 - 1.22)	
Chemotherapy[2]		0.02		0.12		0.69		0.34
No								
Q1	1.00 (Ref)		1.00 (Ref)		1.00 (Ref)		1.00 (Ref)	
Q2	1.23 (0.81 - 1.87)		1.34 (0.76 - 2.35)		1.13 (0.69 - 1.84)		1.13 (0.77 - 1.68)	
Q3	1.25 (0.83 - 1.87)		1.15 (0.65 - 2.02)		1.17 (0.72 - 1.89)		1.22 (0.84 - 1.79)	
Q4	1.47 (0.99 - 2.17)		1.32 (0.75 - 2.30)		1.04 (0.64 - 1.69)		1.20 (0.82 - 1.75)	
Linear	1.19 (0.97 - 1.47)		1.15 (0.87 - 1.51)		1.08 (0.86 - 1.37)		1.15 (0.94 - 1.40)	

Yes								
Q1	1.00 (Ref)		1.00 (Ref)		1.00 (Ref)		1.00 (Ref)	
Q2	0.98 (0.66 - 1.45)		0.94 (0.59 - 1.50)		1.27 (0.83 - 1.95)		1.22 (0.83 - 1.78)	
Q3	0.75 (0.50 - 1.15)		0.65 (0.40 - 1.07)		0.97 (0.63 - 1.50)		0.89 (0.60 - 1.32)	
Q4	0.68 (0.44 - 1.04)		0.60 (0.36 - 1.01)		1.00 (0.64 - 1.56)		0.92 (0.61 - 1.37)	
Linear	0.80 (0.60 - 1.06)		0.74 (0.53 - 1.04)		1.01 (0.77 - 1.33)		0.95 (0.74 - 1.22)	
Stage		0.55		0.26		0.42		0.43
I								
Q1	1.00 (Ref)		1.00 (Ref)		1.00 (Ref)		1.00 (Ref)	
Q2	0.76 (0.38 - 1.53)		0.28 (0.05 - 1.49)		0.42 (0.14 - 1.27)		0.72 (0.37 - 1.39)	
Q3	1.09 (0.57 - 2.05)		0.22 (0.03 - 1.83)		0.58 (0.19 - 1.74)		1.14 (0.63 - 2.05)	
Q4	0.64 (0.31 - 1.31)		0.30 (0.05 - 1.67)		0.45 (0.13 - 1.49)		0.73 (0.38 - 1.41)	
Linear	0.85 (0.54 - 1.35)		0.52 (0.18 - 1.53)		0.75 (0.35 - 1.64)		0.96 (0.62 - 1.48)	
II or III								
Q1	1.00 (Ref)		1.00 (Ref)		1.00 (Ref)		1.00 (Ref)	
Q2	1.01 (0.76 - 1.34)		1.00 (0.71 - 1.43)		1.11 (0.81 - 1.53)		1.08 (0.82 - 1.41)	
Q3	0.98 (0.73 - 1.30)		0.85 (0.59 - 1.23)		1.08 (0.79 - 1.48)		1.06 (0.81 - 1.39)	
Q4	1.00 (0.75 - 1.32)		0.82 (0.57 - 1.18)		0.98 (0.71 - 1.36)		1.03 (0.79 - 1.35)	

Linear	1.01 (0.85 - 1.20)		0.92 (0.73 - 1.15)		1.02 (0.84 - 1.24)		1.06 (0.85 - 1.32)	
BMI		0.28		0.56		0.60		0.28
<25								
Q1	1.00 (Ref)		1.00 (Ref)		1.00 (Ref)		1.00 (Ref)	
Q2	0.74 (0.50 - 1.11)		0.75 (0.43 - 1.32)		0.98 (0.58 - 1.67)		0.75 (0.51 - 1.12)	
Q3	0.88 (0.59 - 1.33)		0.82 (0.46 - 1.46)		1.19 (0.71 - 2.01)		1.00 (0.67 - 1.48)	
Q4	0.81 (0.54 - 1.21)		0.73 (0.41 - 1.30)		0.90 (0.52 - 1.56)		0.81 (0.54 - 1.21)	
Linear	0.99 (0.81 - 1.21)		0.96 (0.73 - 1.25)		1.00 (0.78 - 1.28)		1.01 (0.83 - 1.23)	
>=25								
Q1	1.00 (Ref)		1.00 (Ref)		1.00 (Ref)		1.00 (Ref)	
Q2	1.29 (0.91 - 1.82)		1.26 (0.81 - 1.94)		1.17 (0.80 - 1.70)		1.28 (0.93 - 1.76)	
Q3	1.12 (0.79 - 1.58)		0.76 (0.48 - 1.21)		0.90 (0.62 - 1.31)		1.12 (0.82 - 1.55)	
Q4	1.15 (0.82 - 1.62)		0.89 (0.56 - 1.40)		1.03 (0.71 - 1.51)		1.18 (0.86 - 1.63)	
Linear	1.06 (0.86 - 1.31)		0.93 (0.72 - 1.22)		1.07 (0.84 - 1.35)		1.13 (0.93 - 1.37)	
Diabetes		0.31		0.89		0.88		0.55
No								
Q1	1.00 (Ref)		1.00 (Ref)		1.00 (Ref)		1.00 (Ref)	
Q2	1.01 (0.75 - 1.36)		0.97 (0.65 - 1.43)		1.08 (0.77 - 1.53)		1.05 (0.79 - 1.39)	

Q3	0.98 (0.73 - 1.31)	0.79 (0.53 - 1.17)	1.05 (0.75 - 1.48)	1.08 (0.82 - 1.42)	
Q4	0.88 (0.64 - 1.19)	0.79 (0.52 - 1.20)	0.95 (0.66 - 1.37)	0.93 (0.70 - 1.25)	
Linear	1.00 (0.85 - 1.18)	0.97 (0.78 - 1.20)	1.05 (0.86 - 1.29)	1.06 (0.90 - 1.24)	
Yes					
Q1	1.00 (Ref)	1.00 (Ref)	1.00 (Ref)	1.00 (Ref)	
Q2	0.87 (0.49 - 1.56)	0.99 (0.48 - 2.05)	1.02 (0.53 - 1.99)	0.96 (0.55 - 1.68)	
Q3	0.95 (0.53 - 1.72)	0.66 (0.28 - 1.54)	0.73 (0.36 - 1.49)	0.95 (0.55 - 1.67)	
Q4	1.32 (0.78 - 2.21)	0.85 (0.41 - 1.76)	0.92 (0.49 - 1.75)	1.21 (0.73 - 2.00)	
Linear	1.07 (0.79 - 1.45)	0.80 (0.52 - 1.23)	0.92 (0.64 - 1.32)	1.07 (0.81 - 1.43)	
CVD	0.21	0.30	0.18	0.53	
No					
Q1	1.00 (Ref)	1.00 (Ref)	1.00 (Ref)	1.00 (Ref)	
Q2	0.97 (0.72 - 1.32)	0.91 (0.62 - 1.35)	1.00 (0.71 - 1.40)	1.00 (0.75 - 1.34)	
Q3	0.85 (0.62 - 1.18)	0.63 (0.40 - 0.97)	0.91 (0.64 - 1.29)	0.94 (0.70 - 1.27)	
Q4	0.84 (0.61 - 1.16)	0.78 (0.52 - 1.18)	1.03 (0.72 - 1.46)	0.95 (0.71 - 1.29)	
Linear	0.95 (0.80 - 1.13)	0.90 (0.71 - 1.14)	1.08 (0.88 - 1.34)	1.05 (0.88 - 1.25)	
Yes					
Q1	1.00 (Ref)	1.000(Ref)	1.00 (Ref)	1.00 (Ref)	

Q2	1.16 (0.68 - 1.98)	1.59 (0.76 - 3.30)	1.83 (0.93 - 3.59)	1.28 (0.77 - 2.14)
Q3	1.37 (0.86 - 2.20)	1.28 (0.65 - 2.55)	1.53 (0.82 - 2.87)	1.55 (0.98 - 2.43)
Q4	1.48 (0.93 - 2.36)	1.01 (0.49 - 2.09)	0.90 (0.45 - 1.80)	1.33 (0.83 - 2.11)
Linear	1.18 (0.92 - 1.53)	0.97 (0.68 - 1.39)	0.96 (0.69 - 1.33)	1.15 (0.89 - 1.48)

1. Late-entry models were used after adjusting for age, gender, stage, cancer site, BMI, education, physical activity, screening detected tumor, chemotherapy, diabetes, CVD, constipation, interval between chemotherapy and blood drawn, interval between surgery and blood drawn.
2. Analyses were restricted to stage II-III CRC patients.

Abbreviation: HR: hazard ratio; CI: confidence intervals; p_{int}: p for interaction; Ref.: Reference; BMI: body mass index; CVD: cardiovascular disease.

Table S3. Association between luteolin and colorectal cancer prognosis according to different subgroups[1.]

	overall mortality		CRC-specific mortality		CRC-recurrence		Disease-free survival	
	HR (95% CI)	p_{int}	HR (95% CI)	p_{int}	HR (95% CI)	p_{int}	HR (95% CI)	p_{int}
Age		0.59		0.59		0.28		0.54
<70								
Q1	1.00 (Ref)		1.00 (Ref)		1.00 (Ref)		1.00 (Ref)	
Q2	0.92 (0.60 - 1.42)		0.81 (0.48 - 1.37)		0.66 (0.43 - 1.02)		0.75 (0.51 - 1.10)	
Q3	0.77 (0.49 - 1.19)		0.54 (0.31 - 0.95)		0.64 (0.42 - 0.99)		0.79 (0.54 - 1.15)	
Q4	1.00 (0.65 - 1.53)		0.84 (0.50 - 1.42)		0.73 (0.47 - 1.12)		0.86 (0.58 - 1.26)	
Linear	0.92 (0.61 - 1.39)		0.73 (0.44 - 1.22)		0.69 (0.45 - 1.05)		0.82 (0.57 - 1.19)	
>70								
Q1	1.00 (Ref)		1.00 (Ref)		1.00 (Ref)		1.00 (Ref)	
Q2	1.22 (0.87 - 1.70)		1.11 (0.69 - 1.78)		0.98 (0.63 - 1.52)		1.10 (0.79 - 1.53)	
Q3	1.25 (0.89 - 1.75)		0.95 (0.58 - 1.57)		0.90 (0.57 - 1.43)		1.11 (0.79 - 1.56)	
Q4	1.31 (0.95 - 1.81)		1.22 (0.76 - 1.94)		1.38 (0.91 - 2.09)		1.38 (1.01 - 1.89)	
Linear	1.24 (0.93 - 1.65)		1.16 (0.76 - 1.77)		1.32 (0.90 - 1.95)		1.29 (0.97 - 1.71)	
Gender		0.39		0.42		0.17		0.20
Male								
Q1	1.00 (Ref)		1.00 (Ref)		1.00 (Ref)		1.00 (Ref)	

Q2	1.18 (0.84 - 1.65)	1.09 (0.68 - 1.77)	0.82 (0.54 - 1.23)	0.93 (0.68 - 1.28)
Q3	1.20 (0.85 - 1.69)	0.94 (0.57 - 1.57)	0.98 (0.66 - 1.46)	1.07 (0.78 - 1.46)
Q4	1.15 (0.83 - 1.60)	0.98 (0.60 - 1.60)	0.96 (0.65 - 1.42)	1.04 (0.76 - 1.41)
Linear	1.08 (0.79 - 1.47)	0.96 (0.60 - 1.55)	0.94 (0.64 - 1.38)	0.96 (0.71 - 1.29)
Female				
Q1	1.00 (Ref)	1.00 (Ref)	1.00 (Ref)	1.00 (Ref)
Q2	0.94 (0.62 - 1.43)	0.79 (0.48 - 1.32)	0.82 (0.51 - 1.30)	0.94 (0.62 - 1.42)
Q3	0.84 (0.54 - 1.30)	0.62 (0.35 - 1.09)	0.56 (0.33 - 0.94)	0.77 (0.50 - 1.18)
Q4	1.27 (0.84 - 1.90)	1.10 (0.67 - 1.80)	1.10 (0.70 - 1.72)	1.34 (0.91 - 1.98)
Linear	1.18 (0.84 - 1.66)	0.94 (0.60 - 1.46)	1.06 (0.71 - 1.59)	1.29 (0.93 - 1.79)
Cancer site	0.91	0.54	0.33	0.69
Proximal colon				
Q1	1.00 (Ref)	1.00 (Ref)	1.00 (Ref)	1.00 (Ref)
Q2	1.05 (0.68 - 1.62)	1.13 (0.63 - 2.03)	0.93 (0.55 - 1.58)	0.97 (0.64 - 1.48)
Q3	0.83 (0.52 - 1.33)	0.54 (0.26 - 1.11)	0.51 (0.27 - 0.94)	0.75 (0.48 - 1.17)
Q4	1.08 (0.69 - 1.68)	0.89 (0.46 - 1.74)	1.10 (0.64 - 1.91)	1.13 (0.74 - 1.72)
Linear	1.00 (0.68 - 1.47)	0.79 (0.43 - 1.44)	1.12 (0.67 - 1.88)	1.07 (0.73 - 1.56)
Distal colon				

Q1	1.00 (Ref)	1.00 (Ref)	1.00 (Ref)	1.00 (Ref)
Q2	0.96 (0.52 - 1.76)	0.61 (0.25 - 1.49)	0.67 (0.32 - 1.38)	0.85 (0.48 - 1.49)
Q3	1.26 (0.72 - 2.20)	1.16 (0.54 - 2.48)	1.38 (0.73 - 2.61)	1.40 (0.84 - 2.33)
Q4	1.15 (0.66 - 2.02)	1.11 (0.52 - 2.36)	1.21 (0.65 - 2.25)	1.21 (0.73 - 2.01)
Linear	1.28 (0.74 - 2.21)	1.03 (0.47 - 2.28)	0.94 (0.52 - 1.72)	1.14 (0.70 - 1.85)
Rectal				
Q1	1.00 (Ref)	1.00 (Ref)	1.00 (Ref)	1.00 (Ref)
Q2	1.25 (0.84 - 1.86)	0.97 (0.59 - 1.61)	0.80 (0.52 - 1.25)	0.95 (0.65 - 1.39)
Q3	1.10 (0.73 - 1.66)	0.74 (0.42 - 1.28)	0.82 (0.52 - 1.29)	0.92 (0.62 - 1.35)
Q4	1.38 (0.93 - 2.04)	1.15 (0.70 - 1.87)	0.94 (0.61 - 1.46)	1.16 (0.80 - 1.67)
Linear	1.17 (0.83 - 1.66)	1.05 (0.66 - 1.66)	0.94 (0.62 - 1.42)	1.07 (0.76 - 1.51)
Chemotherapy[2]	0.16	0.36	0.03	0.01
Without				
Q1	1.00 (Ref)	1.00 (Ref)	1.00 (Ref)	1.00 (Ref)
Q2	1.27 (0.85 - 1.90)	1.05 (0.60 - 1.83)	1.17 (0.71 - 1.94)	1.25 (0.84 - 1.84)
Q3	1.27 (0.85 - 1.89)	0.94 (0.52 - 1.68)	1.19 (0.71 - 2.01)	1.34 (0.90 - 1.98)
Q4	1.43 (0.98 - 2.09)	1.36 (0.82 - 2.26)	1.60 (1.01 - 2.55)	1.53 (1.06 - 2.21)
Linear	1.15 (0.83 - 1.58)	1.13 (0.71 - 1.79)	1.30 (0.86 - 1.96)	1.21 (0.89 - 1.65)

With				
Q1	1.00 (Ref)	1.00 (Ref)	1.00 (Ref)	1.00 (Ref)
Q2	0.79 (0.52 - 1.18)	0.82 (0.52 - 1.32)	0.59 (0.39 - 0.89)	0.61 (0.42 - 0.90)
Q3	0.65 (0.43 - 0.98)	0.55 (0.33 - 0.91)	0.52 (0.34 - 0.80)	0.57 (0.39 - 0.84)
Q4	0.86 (0.57 - 1.29)	0.75 (0.45 - 1.23)	0.67 (0.44 - 1.02)	0.75 (0.52 - 1.10)
Linear	0.95 (0.65 - 1.38)	0.79 (0.50 - 1.25)	0.81 (0.55 - 1.19)	0.88 (0.62 - 1.25)
Stage	0.06	0.21	0.49	0.18
I				
Q1	1.00 (Ref)	1.00 (Ref)	1.00 (Ref)	1.00 (Ref)
Q1	2.44 (1.14 - 5.19)	5.67 (0.59 - 54.2)	1.57 (0.47 - 5.22)	1.73 (0.88 - 3.42)
Q3	2.49 (1.16 - 5.33)	4.73 (0.50 - 44.9)	1.43 (0.43 - 4.70)	1.85 (0.93 - 3.65)
Q4	1.51 (0.70 - 3.26)	2.82 (0.28 - 28.4)	1.26 (0.37 - 4.24)	1.44 (0.73 - 2.84)
Linear	1.31 (0.70 - 2.43)	0.84 (0.19 - 3.65)	0.59 (0.23 - 1.53)	1.15 (0.64 - 2.04)
II or III				
Q1	1.00 (Ref)	1.00 (Ref)	1.00 (Ref)	1.00 (Ref)
Q2	0.92 (0.69 - 1.21)	0.86 (0.61 - 1.22)	0.76 (0.55 - 1.04)	0.81 (0.62 - 1.06)
Q3	0.87 (0.66 - 1.16)	0.65 (0.45 - 0.95)	0.71 (0.52 - 0.99)	0.83 (0.63 - 1.09)
Q4	1.10 (0.84 - 1.44)	0.99 (0.70 - 1.40)	1.01 (0.75 - 1.36)	1.08 (0.83 - 1.39)

Linear	1.07 (0.84 - 1.36)		0.96 (0.70 - 1.31)		1.05 (0.79 - 1.39)		1.09 (0.86 - 1.37)	
BMI		0.54		0.21		0.55		0.50
<25								
Q1	1.00 (Ref)		1.00 (Ref)		1.00 (Ref)		1.00 (Ref)	
Q2	1.26 (0.84 - 1.88)		1.17 (0.66 - 2.07)		0.98 (0.58 - 1.63)		1.11 (0.75 - 1.65)	
Q3	1.20 (0.80 - 1.79)		1.35 (0.76 - 2.41)		1.10 (0.66 - 1.85)		1.10 (0.74 - 1.64)	
Q4	1.08 (0.72 - 1.62)		1.19 (0.67 - 2.13)		1.09 (0.66 - 1.80)		1.08 (0.74 - 1.60)	
Linear	0.98 (0.71 - 1.35)		1.08 (0.66 - 1.75)		1.16 (0.75 - 1.79)		1.06 (0.78 - 1.45)	
≥25								
Q1	1.00 (Ref)		1.00 (Ref)		1.00 (Ref)		1.00 (Ref)	
Q2	1.00 (0.71 - 1.41)		0.84 (0.54 - 1.29)		0.71 (0.49 - 1.04)		0.82 (0.59 - 1.12)	
Q3	0.96 (0.67 - 1.37)		0.55 (0.33 - 0.90)		0.66 (0.45 - 0.98)		0.87 (0.63 - 1.20)	
Q4	1.22 (0.87 - 1.70)		0.90 (0.59 - 1.38)		0.90 (0.63 - 1.30)		1.11 (0.82 - 1.51)	
Linear	1.20 (0.88 - 1.65)		0.80 (0.52 - 1.24)		0.81 (0.56 - 1.18)		1.05 (0.77 - 1.41)	
Diabetes History		0.14		0.67		0.44		0.10
No								
Q1	1.00 (Ref)		1.00 (Ref)		1.00 (Ref)		1.00 (Ref)	
Q2	1.08 (0.80 - 1.47)		0.99 (0.66 - 1.47)		0.81 (0.57 - 1.14)		0.91 (0.68 - 1.21)	

Q3	0.97 (0.71 - 1.32)	0.73 (0.47 - 1.12)	0.73 (0.51 - 1.05)	0.87 (0.65 - 1.16)	
Q4	1.26 (0.94 - 1.68)	1.09 (0.74 - 1.62)	1.06 (0.76 - 1.48)	1.20 (0.92 - 1.58)	
Linear	1.15 (0.88 - 1.49)	0.97 (0.67 - 1.42)	1.05 (0.76 - 1.46)	1.16 (0.90 - 1.50)	
Yes					
Q1	1.00 (Ref)	1.00 (Ref)	1.00 (Ref)	1.00 (Ref)	
Q2	1.41 (0.83 - 2.40)	1.01 (0.49 - 2.06)	0.78 (0.42 - 1.47)	1.06 (0.64 - 1.74)	
Q3	1.62 (0.95 - 2.76)	1.17 (0.54 - 2.52)	1.16 (0.61 - 2.20)	1.45 (0.87 - 2.42)	
Q4	1.09 (0.63 - 1.89)	0.90 (0.43 - 1.89)	0.75 (0.40 - 1.42)	0.89 (0.53 - 1.50)	
Linear	1.11 (0.67 - 1.82)	0.94 (0.48 - 1.87)	0.72 (0.40 - 1.29)	0.87 (0.55 - 1.40)	
CVD	0.14	0.79	0.77		0.25
No					
Q1	1.00 (Ref)	1.00 (Ref)	1.00 (Ref)	1.00 (Ref)	
Q2	0.93 (0.67 - 1.28)	0.91 (0.61 - 1.37)	0.78 (0.55 - 1.11)	0.81 (0.60 - 1.09)	
Q3	0.99 (0.71 - 1.37)	0.76 (0.48 - 1.18)	0.81 (0.57 - 1.16)	0.91 (0.67 - 1.23)	
Q4	1.13 (0.83 - 1.53)	1.04 (0.70 - 1.57)	1.02 (0.73 - 1.43)	1.10 (0.82 - 1.46)	
Linear	1.09 (0.81 - 1.48)	0.93 (0.61 - 1.40)	0.95 (0.67 - 1.34)	1.05 (0.79 - 1.40)	
Yes					
Q1	1.00 (Ref)	1.00 (Ref)	1.00 (Ref)	1.00 (Ref)	

Q2	1.77 (1.11 - 2.82)	1.32 (0.67 - 2.59)	1.02 (0.53 - 1.94)	1.45 (0.91 - 2.30)
Q3	1.23 (0.76 - 1.98)	0.78 (0.38 - 1.59)	0.79 (0.41 - 1.50)	1.13 (0.71 - 1.80)
Q4	1.27 (0.80 - 2.01)	0.99 (0.51 - 1.91)	0.99 (0.53 - 1.82)	1.20 (0.76 - 1.89)
Linear	1.15 (0.80 - 1.64)	0.98 (0.56 - 1.71)	1.03 (0.62 - 1.71)	1.13 (0.80 - 1.61)

1. Late-entry models were used after adjusting for age, gender, stage, cancer site, BMI, education, physical activity, screening detected tumor, chemotherapy, diabetes, CVD, constipation, interval between chemotherapy and blood drawn, interval between surgery and blood drawn.
2. Analyses were restricted to stage II-III CRC patients.

Abbreviation: HR: hazard ratio; CI: confidence intervals; p_{int}: p for interaction; Ref.: Reference; BMI: body mass index; CVD: cardiovascular disease.

Table S4. Association of serum genistein and colorectal cancer prognosis by timing of blood drawn[1.]

	Overall mortality		CRC-specific mortality		CRC-recurrence		Disease-free survival	
	HR (95% CI)	p_{int}	HR (95% CI)	p_{int}	HR (95% CI)	p_{int}	HR (95% CI)	p_{int}
Diagnosis (month)[2]		0.60		0.22		0.49		0.88
<1								
Q1	1.00 (Ref)		1.00 (Ref)		1.00 (Ref)		1.00 (Ref)	
Q2	1.12 (0.77 - 1.64)		1.42 (0.85 - 2.39)		1.32 (0.84 - 2.08)		1.11 (0.77 - 1.59)	
Q3	0.93 (0.63 - 1.38)		0.89 (0.51 - 1.57)		1.12 (0.70 - 1.78)		1.07 (0.74 - 1.53)	
Q4	1.10 (0.75 - 1.60)		1.17 (0.69 - 1.98)		1.13 (0.71 - 1.80)		1.07 (0.74 - 1.53)	
Linear	1.00 (0.79 - 1.27)		1.00 (0.74 - 1.35)		1.04 (0.79 - 1.36)		1.11 (0.80 - 1.52)	
1-6								
Q1	1.00 (Ref)		1.00 (Ref)		1.00 (Ref)		1.00 (Ref)	
Q2	0.73 (0.41 - 1.32)		0.45 (0.20 - 1.00)		0.68 (0.35 - 1.31)		0.79 (0.46 - 1.35)	
Q3	1.11 (0.64 - 1.93)		0.49 (0.23 - 1.05)		0.71 (0.39 - 1.29)		0.95 (0.57 - 1.58)	
Q4	0.69 (0.37 - 1.28)		0.38 (0.17 - 0.87)		0.76 (0.40 - 1.44)		0.88 (0.51 - 1.54)	
Linear	1.00 (0.75 - 1.33)		0.88 (0.62 - 1.25)		1.09 (0.79 - 1.49)		1.11 (0.84 - 1.46)	
>6								
Q1	1.00 (Ref)		1.00 (Ref)		1.00 (Ref)		1.00 (Ref)	
Q2	0.95 (0.58 - 1.56)		0.76 (0.40 - 1.46)		1.02 (0.56 - 1.85)		1.08 (0.67 - 1.75)	

Q3	1.07 (0.66 - 1.75)	0.91 (0.48 - 1.72)	1.27 (0.72 - 2.27)	1.30 (0.81 - 2.09)
Q4	1.11 (0.69 - 1.78)	0.83 (0.43 - 1.58)	1.04 (0.57 - 1.88)	1.16 (0.72 - 1.86)
Linear	1.08 (0.82 - 1.42)	0.95 (0.64 - 1.40)	1.12 (0.78 - 1.61)	1.18 (0.89 - 1.57)
Chemotherapy[3]				
Before chemotherapy	0.75	0.25	0.84	0.82
Q1	1.00 (Ref)	1.00 (Ref)	1.00 (Ref)	1.00 (Ref)
Q2	1.08 (0.61 - 1.93)	1.64 (0.86 - 3.15)	1.41 (0.79 - 2.53)	1.07 (0.62 - 1.83)
Q3	0.74 (0.39 - 1.39)	0.73 (0.35 - 1.55)	0.86 (0.46 - 1.62)	0.79 (0.44 - 1.40)
Q4	0.47 (0.23 - 0.96)	0.56 (0.25 - 1.26)	0.76 (0.39 - 1.48)	0.63 (0.34 - 1.17)
Linear	0.60 (0.38 - 0.94)	0.62 (0.37 - 1.02)	0.82 (0.53 - 1.26)	0.77 (0.52 - 1.16)
After chemotherapy				
Q1	1.00 (Ref)	1.00 (Ref)	1.00 (Ref)	1.00 (Ref)
Q2	0.97 (0.56 - 1.71)	0.58 (0.29 - 1.16)	1.19 (0.62 - 2.26)	1.46 (0.83 - 2.58)
Q3	0.80 (0.45 - 1.43)	0.60 (0.31 - 1.17)	1.11 (0.59 - 2.08)	1.06 (0.59 - 1.90)
Q4	0.80 (0.45 - 1.43)	0.57 (0.29 - 1.13)	1.25 (0.67 - 2.36)	1.26 (0.70 - 2.24)
Linear	0.89 (0.62 - 1.27)	0.83 (0.53 - 1.32)	1.15 (0.80 - 1.65)	1.06 (0.76 - 1.47)

1. Late-entry models were used after adjusting for age, gender, stage, cancer site, BMI, education, physical activity, screening detected tumor, chemotherapy, diabetes, CVD, constipation, interval between chemotherapy and blood drawn, interval between surgery and blood drawn.
2. Interval between diagnosis and blood drawn.
3. Timing of blood drawn with respect to chemotherapy. Analyses were restricted to stage II-III CRC patients who underwent chemotherapy.

Abbreviation: HR: hazard ratio; CI: confidence intervals; p_{int}: p for interaction; Ref.: Reference.

Table S5. Association of serum luteolin and colorectal cancer prognosis by timing of blood drawn[1.]

	Overall mortality		CRC-specific mortality		CRC-recurrence		Disease-free survival	
	HR (95% CI)	p_{int}	HR (95% CI)	p_{int}	HR (95% CI)	p_{int}	HR (95% CI)	p_{int}
Diagnosis (month)[2]		0.12		0.04		0.27		0.50
<1								
Q1	1.00 (Ref)		1.00 (Ref)		1.00 (Ref)		1.00 (Ref)	
Q2	1.20 (0.82 - 1.76)		1.19 (0.71 - 1.98)		0.88 (0.57 - 1.38)		0.97 (0.68 - 1.39)	
Q3	1.09 (0.73 - 1.63)		0.93 (0.53 - 1.65)		0.84 (0.52 - 1.35)		0.98 (0.68 - 1.42)	
Q4	1.41 (0.99 - 2.01)		1.47 (0.90 - 2.40)		1.14 (0.75 - 1.73)		1.20 (0.86 - 1.68)	
Linear	1.26 (0.93 - 1.73)		1.14 (0.73 - 1.77)		1.04 (0.71 - 1.53)		1.14 (0.85 - 1.53)	
1-6								
Q1	1.00 (Ref)		1.00 (Ref)		1.00 (Ref)		1.00 (Ref)	
Q2	0.78 (0.45 - 1.37)		0.40 (0.18 - 0.89)		0.49 (0.26 - 0.95)		0.71 (0.42 - 1.21)	
Q3	0.59 (0.33 - 1.06)		0.23 (0.10 - 0.52)		0.45 (0.24 - 0.85)		0.62 (0.36 - 1.05)	
Q4	0.76 (0.41 - 1.41)		0.58 (0.26 - 1.26)		1.00 (0.53 - 1.88)		0.99 (0.56 - 1.73)	
Linear	0.77 (0.48 - 1.24)		0.48 (0.24 - 0.97)		1.07 (0.57 - 2.02)		1.02 (0.63 - 1.67)	
>6								
Q1	1.00 (Ref)		1.00 (Ref)		1.00 (Ref)		1.00 (Ref)	
Q2	1.40 (0.84 - 2.32)		1.33 (0.70 - 2.53)		1.04 (0.58 - 1.85)		1.18 (0.72 - 1.92)	

Q3	1.84 (1.14 - 2.97)	1.35 (0.71 - 2.57)	1.13 (0.64 - 2.01)	1.47 (0.92 - 2.35)
Q4	1.22 (0.76 - 1.98)	0.89 (0.46 - 1.71)	0.90 (0.51 - 1.58)	1.22 (0.77 - 1.93)
Linear	1.19 (0.76 - 1.86)	1.04 (0.57 - 1.92)	0.82 (0.46 - 1.46)	1.05 (0.66 - 1.66)
Chemotherapy[3]	0.27	0.71	0.16	0.22
Before chemotherapy				
Q1	1.00 (Ref)	1.00 (Ref)	1.00 (Ref)	1.00 (Ref)
Q2	0.72 (0.40 - 1.29)	0.93 (0.48 - 1.80)	0.70 (0.39 - 1.27)	0.63 (0.36 - 1.09)
Q3	0.37 (0.18 - 0.76)	0.46 (0.20 - 1.03)	0.50 (0.26 - 0.98)	0.46 (0.25 - 0.86)
Q4	0.76 (0.41 - 1.40)	0.82 (0.39 - 1.70)	0.73 (0.39 - 1.38)	0.72 (0.41 - 1.29)
Linear	0.66 (0.35 - 1.24)	0.69 (0.34 - 1.38)	0.76 (0.41 - 1.38)	0.74 (0.42 - 1.30)
After chemotherapy				
Q1	1.00 (Ref)	1.00 (Ref)	1.00 (Ref)	1.00 (Ref)
Q2	0.83 (0.46 - 1.50)	0.71 (0.35 - 1.42)	0.51 (0.28 - 0.94)	0.62 (0.36 - 1.07)
Q3	0.94 (0.55 - 1.62)	0.64 (0.32 - 1.25)	0.55 (0.31 - 0.97)	0.67 (0.41 - 1.11)
Q4	1.00 (0.58 - 1.73)	0.68 (0.34 - 1.35)	0.66 (0.37 - 1.16)	0.79 (0.48 - 1.32)
Linear	1.21 (0.73 - 2.01)	0.88 (0.46 - 1.67)	0.84 (0.48 - 1.45)	0.98 (0.59 - 1.60)

1. Late-entry models were used after adjusting for age, gender, stage, cancer site, BMI, education, physical activity, screening detected tumor, chemotherapy, diabetes, CVD, constipation, interval between chemotherapy and blood drawn, interval between surgery and blood drawn.
2. Interval between diagnosis and blood drawn.
3. Timing of blood drawn with respect to chemotherapy. Analyses were restricted to stage II-III CRC patients who underwent chemotherapy.

Abbreviation: HR: hazard ratio; CI: confidence intervals; p_{int}: p for interaction; Ref.: Reference.

Table S6. Association between genistein and colorectal cancer prognosis by tumoral molecular characterization[1]

	Overall mortality		CRC-specific mortality		CRC-recurrence		Disease-free survival	
	HR (95% CI)	p_{int}	HR (95% CI)	p_{int}	HR (95% CI)	p_{int}	HR (95% CI)	p_{int}
KRAS[2]		0.51		0.55		0.87		0.64
Wild								
Q1	1.00 (Ref)		1.00 (Ref)		1.00 (Ref)		1.00 (Ref)	
Q2	0.91 (0.63 - 1.31)		0.94 (0.58 - 1.53)		1.03 (0.67 - 1.59)		0.92 (0.64 - 1.30)	
Q3	0.95 (0.66 - 1.38)		0.81 (0.49 - 1.33)		0.91 (0.58 - 1.43)		0.98 (0.69 - 1.40)	
Q4	0.92 (0.63 - 1.34)		0.77 (0.46 - 1.29)		0.84 (0.53 - 1.33)		0.89 (0.62 - 1.28)	
Linear	0.94 (0.74 - 1.18)		0.88 (0.64 - 1.21)		0.93 (0.69 - 1.25)		0.96 (0.77 - 1.21)	
Mutant								
Q1	1.00 (Ref)		1.00 (Ref)		1.00 (Ref)		1.00 (Ref)	
Q2	1.27 (0.74 - 2.16)		1.39 (0.71 - 2.73)		1.31 (0.72 - 2.36)		1.32 (0.81 - 2.17)	
Q3	1.26 (0.72 - 2.19)		0.72 (0.33 - 1.59)		1.12 (0.61 - 2.06)		1.35 (0.81 - 2.23)	
Q4	0.97 (0.56 - 1.69)		0.77 (0.37 - 1.63)		1.04 (0.57 - 1.89)		1.14 (0.69 - 1.88)	
Linear	1.10 (0.87 - 1.40)		0.95 (0.70 - 1.29)		1.11 (0.84 - 1.46)		1.18 (0.94 - 1.49)	
CIMP[3]		0.03		0.29		0.89		0.42
Low/Negative								
Q1	1.00 (Ref)		1.00 (Ref)		1.00 (Ref)		1.00 (Ref)	

Q2	0.99 (0.72 - 1.36)	1.01 (0.67 - 1.51)	1.09 (0.76 - 1.55)	1.05 (0.78 - 1.41)
Q3	0.89 (0.64 - 1.24)	0.77 (0.50 - 1.18)	1.02 (0.71 - 1.46)	1.01 (0.75 - 1.37)
Q4	0.93 (0.68 - 1.29)	0.82 (0.53 - 1.25)	0.99 (0.69 - 1.44)	1.00 (0.74 - 1.36)
Linear	1.01 (0.85 - 1.19)	0.96 (0.78 - 1.18)	1.06 (0.88 - 1.28)	1.07 (0.91 - 1.25)
High				
Q1	1.00 (Ref)	1.00 (Ref)	1.00 (Ref)	1.00 (Ref)
Q2	2.42 (0.98 - 5.98)	1.85 (0.52 - 6.60)	1.37 (0.45 - 4.15)	1.67 (0.71 - 3.90)
Q3	3.48 (1.44 - 8.40)	1.48 (0.40 - 5.53)	0.93 (0.29 - 3.02)	2.22 (0.96 - 5.11)
Q4	2.05 (0.83 - 5.08)	0.88 (0.22 - 3.60)	0.90 (0.29 - 2.79)	1.51 (0.66 - 3.46)
Linear	1.23 (0.76 - 2.01)	1.07 (0.43 - 2.62)	1.21 (0.60 - 2.46)	1.22 (0.78 - 1.92)
ESR2[4]	0.65	0.99	0.92	0.58
Negative				
Q1	1.00 (Ref)	1.00 (Ref)	1.00 (Ref)	1.00 (Ref)
Q2	1.05 (0.66 - 1.68)	1.02 (0.57 - 1.84)	1.13 (0.66 - 1.93)	1.10 (0.71 - 1.71)
Q3	0.99 (0.62 - 1.59)	0.72 (0.39 - 1.34)	1.02 (0.60 - 1.73)	1.04 (0.67 - 1.61)
Q4	1.15 (0.73 - 1.81)	0.81 (0.44 - 1.50)	1.13 (0.66 - 1.91)	1.21 (0.78 - 1.86)
Linear	1.12 (0.85 - 1.49)	0.89 (0.60 - 1.32)	1.12 (0.81 - 1.54)	1.19 (0.91 - 1.54)
Positive				

Q1	1.00 (Ref)	1.00 (Ref)	1.00 (Ref)	1.00 (Ref)
Q2	1.03 (0.67 - 1.58)	1.17 (0.65 - 2.11)	1.16 (0.70 - 1.91)	1.01 (0.68 - 1.49)
Q3	0.92 (0.59 - 1.45)	0.67 (0.35 - 1.31)	0.89 (0.52 - 1.54)	1.02 (0.68 - 1.54)
Q4	0.84 (0.53 - 1.32)	0.91 (0.49 - 1.71)	0.95 (0.55 - 1.63)	0.87 (0.57 - 1.32)
Linear	0.98 (0.79 - 1.22)	0.99 (0.75 - 1.31)	1.07 (0.83 - 1.37)	1.04 (0.85 - 1.28)

1. Late-entry models were used after adjusting for age, gender, stage, cancer site, BMI, education, physical activity, screening detected tumor, chemotherapy, diabetes, CVD, constipation, interval between chemotherapy and blood drawn, interval between surgery and blood drawn.
2. Data available for 1584 patients.
3. Data available for 1487 patients.
4. Data available for 1389 patients,

Abbreviation: HR: hazard ratio; CI: confidence intervals; p_{int}: p for interaction; Ref.: Reference; CIMP: CpG island methylator phenotype; ESR2: estrogen receptor beta.

Table S7. Association between luteolin and colorectal cancer prognosis by cancer subtype[1.]

	Overall mortality		CRC-specific mortality		CRC-recurrence		Disease-free survival	
	HR (95% CI)	p_{int}	HR (95% CI)	p_{int}	HR (95% CI)	p_{int}	HR (95% CI)	p_{int}
KRAS[2]		0.61		0.99		0.66		0.66
Wild								
Q1	1.00 (Ref)		1.00 (Ref)		1.00 (Ref)		1.00 (Ref)	
Q2	0.92 (0.63 - 1.33)		1.09 (0.67 - 1.77)		0.88 (0.57 - 1.34)		0.81 (0.57 - 1.15)	
Q3	0.88 (0.61 - 1.26)		0.73 (0.44 - 1.21)		0.73 (0.47 - 1.14)		0.81 (0.57 - 1.15)	
Q4	1.08 (0.76 - 1.55)		0.95 (0.57 - 1.59)		0.86 (0.55 - 1.34)		1.02 (0.72 - 1.43)	
Linear	1.14 (0.81 - 1.60)		0.93 (0.57 - 1.51)		0.86 (0.56 - 1.31)		1.03 (0.75 - 1.43)	
Mutant								
Q1	1.00 (Ref)		1.00 (Ref)		1.00 (Ref)		1.00 (Ref)	
Q2	1.11 (0.66 - 1.86)		0.96 (0.49 - 1.87)		0.86 (0.48 - 1.54)		1.03 (0.64 - 1.66)	
Q3	1.30 (0.76 - 2.21)		0.79 (0.37 - 1.70)		0.77 (0.41 - 1.46)		1.07 (0.64 - 1.77)	
Q4	1.07 (0.63 - 1.81)		0.92 (0.47 - 1.83)		1.21 (0.70 - 2.09)		1.28 (0.80 - 2.04)	
Linear	0.83 (0.52 - 1.31)		0.73 (0.39 - 1.34)		1.12 (0.66 - 1.92)		1.07 (0.70 - 1.65)	
CIMP[3]		0.58		0.28		0.13		0.47
Negative/ Low								
Q1	1.00 (Ref)		1.00 (Ref)		1.00 (Ref)		1.00 (Ref)	

Q2	1.04 (0.76 - 1.43)	1.12 (0.76 - 1.67)	0.95 (0.67 - 1.35)	0.92 (0.68 - 1.23)
Q3	1.01 (0.73 - 1.40)	0.85 (0.55 - 1.32)	0.89 (0.62 - 1.29)	0.94 (0.69 - 1.28)
Q4	1.08 (0.80 - 1.48)	1.02 (0.67 - 1.54)	1.07 (0.75 - 1.51)	1.10 (0.83 - 1.46)
Linear	1.04 (0.79 - 1.38)	0.97 (0.67 - 1.41)	1.09 (0.79 - 1.50)	1.07 (0.83 - 1.39)
High				
Q1	1.00 (Ref)	1.00 (Ref)	1.00 (Ref)	1.00 (Ref)
Q2	1.32 (0.59 - 2.92)	0.60 (0.18 - 1.98)	0.51 (0.18 - 1.43)	1.16 (0.54 - 2.48)
Q3	1.23 (0.57 - 2.66)	0.36 (0.09 - 1.40)	0.26 (0.08 - 0.84)	0.94 (0.44 - 1.99)
Q4	2.76 (1.25 - 6.10)	1.81 (0.53 - 6.22)	1.38 (0.48 - 3.98)	2.52 (1.18 - 5.41)
Linear	1.76 (0.80 - 3.85)	0.73 (0.21 - 2.54)	0.51 (0.19 - 1.39)	1.30 (0.61 - 2.73)
ESR2 expression [4]	0.80	0.15	0.25	0.90
Negative				
Q1	1.00 (Ref)	1.00 (Ref)	1.00 (Ref)	1.00 (Ref)
Q2	1.14 (0.73 - 1.79)	1.00 (0.57 - 1.74)	0.94 (0.57 - 1.54)	1.07 (0.70 - 1.63)
Q3	0.83 (0.51 - 1.34)	0.39 (0.18 - 0.84)	0.66 (0.38 - 1.14)	0.88 (0.56 - 1.37)
Q4	1.32 (0.87 - 1.99)	0.82 (0.47 - 1.46)	0.86 (0.52 - 1.40)	1.21 (0.81 - 1.79)
Linear	1.14 (0.79 - 1.65)	0.66 (0.40 - 1.11)	0.67 (0.43 - 1.06)	0.97 (0.68 - 1.37)
Positive				

Q1	1.00 (Ref)	1.00 (Ref)	1.00 (Ref)	1.00 (Ref)
Q2	0.99 (0.64 - 1.52)	1.15 (0.62 - 2.13)	0.83 (0.49 - 1.42)	0.84 (0.56 - 1.27)
Q3	0.92 (0.59 - 1.44)	0.87 (0.45 - 1.67)	0.76 (0.44 - 1.31)	0.83 (0.55 - 1.26)
Q4	1.06 (0.69 - 1.65)	1.59 (0.87 - 2.91)	1.57 (0.95 - 2.59)	1.23 (0.83 - 1.83)
Linear	0.88 (0.58 - 1.34)	1.19 (0.68 - 2.08)	1.55 (0.97 - 2.50)	1.11 (0.75 - 1.64)

1. Late-entry models were used after adjusting for age, gender, stage, cancer site, BMI, education, physical activity, screening detected tumor, chemotherapy, diabetes, CVD, constipation, interval between chemotherapy and blood drawn, interval between surgery and blood drawn.
2. Data available for 1476 patients.
   Data available for 1572 patients.
3. Data available for 1380 patients.

Abbreviation: HR: hazard ratio; CI: confidence intervals; p_{int} : p for interaction; Ref.: Reference; CIMP: CpG island methylator phenotype; ESR2: estrogen receptor beta.
